# Supplementary material for: Biodiversity can benefit from climate stabilization despite adverse side effects of land-based mitigation
Source: Nat Commun. 2019 Nov 20;10:5240. doi: 10.1038/s41467-019-13241-y (PMC6868141; doi:10.1038/s41467-019-13241-y)
Supplement: Supplementary file 1 — Supplementary Information [file 41467_2019_13241_MOESM1_ESM.pdf]

Supplementary Information

Biodiversity can benefit from climate stabilization despite adverse side-effects of land-based mitigation

Ohashi et al.

Correspondence to: Haruka Ohashi (oharu0429@gmail.com)

This PDF file includes:

Supplementary Table 1-3

Supplementary Figure 1-9

Supplementary References

Other Supplementary materials for this manuscript include the following:

Supplementary Data

Supplementary Table 1 Relationship of land-use change scenarios, climate change scenarios, and three hypothetical combination of land-use and climate change scenarios (LU, CC, and LUCC).

Climate change sub-scenarios				Land-use change sub-scenarios				
				Current	Mitigation		Baseline	
					SSP1_26W, SSP2_26W, SSP3_34W, SSP4_26W, SSP5_26W		SSP1_Baseline, SSP2_ Baseline, SSP3_ Baseline, SSP4_Baseline, SSP5_Baseline	
Scenario	RCP	Year	GCMs	2005	2050	2070	2050	2070
Current	–	1960 – 1990	–	Current	LU (MIT-2050s)	LU (MIT-2070s)	LU (BL-2050s)	LU (BL-2070s)
Mitigation (MIT)	2.6	2050s	gf he ip mi no	CC (MIT-2050s)	LUCC (MIT-2050s)	–	–	–
		2070s	gf he ip mi no	CC (MIT-2070s)	–	LUCC (MIT-2070s)	–	–
Baseline (BL)	8.5	2050s	gf he ip mi no	CC (BL-2050s)	–	–	LUCC (BL-2050s)	–
		2070s	gf he ip mi no	CC (BL-2070s)	–	–	–	LUCC (BL-2070s)

gf: GFDL-CM3; he: HadGEM2-ES; ip: IPSL-CM5A-LR; mi: MIROC-ESM-CHEM; no: NorESM1-M

Supplementary Table 2 Coverage of modeled species within species on IUCN Red List (with region information)

	Vascular plants			Amphibians*			Reptiles			Birds			Mammals**		
	Modeled	Total	%	Modeled	Total	%	Modeled	Total	%	Modeled	Total	%	Modeled	Total	%
Native region															
North America	233	2631	8.9	151	1035	14.6	217	1140	19	445	881	50.5	284	647	43.9
South America	184	4845	3.8	78	2193	3.6	12	399	3	1068	2401	44.5	127	987	12.9
Europe	62	810	7.7	15	55	27.3	14	70	20	6	22	27.3	14	44	31.8
Africa	185	3443	5.4	56	1030	5.4	27	971	2.8	850	1789	47.5	196	1213	16.2
Asia	175	4922	3.6	45	1301	3.5	15	1357	1.1	612	2247	27.2	62	1481	4.2
Oceania	104	1180	8.8	95	423	22.5	52	446	11.7	312	824	37.9	135	366	36.9
Multiple Region	662	1210	54.7	69	195	35.4	44	247	17.8	1503	2109	71.3	319	585	54.5
Red List category															
CR	4	2150	0.2	1	528	0.2	1	180	0.6	9	217	4.1	3	208	1.4
EN	25	3308	0.8	5	805	0.6	7	361	1.9	31	416	7.5	16	475	3.4
VU	110	5224	2.1	13	653	2	11	402	2.7	81	740	10.9	30	501	6
LR/cd	9	209	4.3	0	0	-	1	2	50	0	0	-	0	0	-
NT or LR/nt	84	1543	5.4	24	400	6	14	316	4.4	181	968	18.7	50	321	15.6
LC or LR/lc	1342	5208	25.8	462	2375	19.5	345	2518	13.7	4494	7871	57.1	1026	3082	33.3
DD	31	1399	2.2	4	1471	0.3	2	851	0.2	0	61	0	12	736	1.6
Total	1605	19041	8.4	509	6232	8.2	381	4630	8.2	4796	10273	46.7	1137	5323	21.4

* Gymnophiona were excluded from calculation. **Sirenians and Cetaceans were excluded from calculation.

Supplementary Table 3. Compiled data for estimating dispersal distance of reptiles.

Species	Order	Family	Dispersal distance (m)	Ref. ID (DD)	Adult body mass (g)	Ref. ID (ABM)
Crocodylus intermedius	Crocodilia	Crocodylidae	5300.0	1	107900.0	13
Crocodylus johnsoni	Crocodilia	Crocodylidae	11750.0	2	19500.0	13
Amphibolurus muricatus	Squamata	Agamidae	67.4	3	3.9	*
Stegonotus cucullatus	Squamata	Colubridae	257.0	4	848.0	13
Diplodactylus conspicillatus	Squamata	Diplodactylidae	35.8	5	3.9	13
Lucasium stenodactylum	Squamata	Diplodactylidae	39.2	5	5.6	13
Rhynchoedura ornata	Squamata	Diplodactylidae	38.3	5	1.8	13
Iguana iguana	Squamata	Iguanidae	425.0	6	1530.0	13
Lacerta agilis	Squamata	Lacertidae	135.0	7	9.9	13
Takydromus tachydromoides	Squamata	Lacertidae	13.0	8	3.3	13
Concinnia queenslandiae	Squamata	Scincidae	29.0	9	1.8	**
Niveoscincus microlepidotus	Squamata	Scincidae	9.4	10	4.0	13
Crotalus horridus	Squamata	Viperidae	840.0	11	1000.0	13
Trachemys scripta	Testudines	Emydidae	734.0	12	1854.0	13
Mauremys japonica	Testudines	Geoemydidae	375.0	12	494.2	13
Mauremys reevesii	Testudines	Geoemydidae	675.0	12	858.0	13

* Estimated from SVL-Mass allometry in Meiri (ref.14); SVL=100mm

** Estimated from SVL-Mass allometry in Meiri (ref.14); SVL=62.1mm

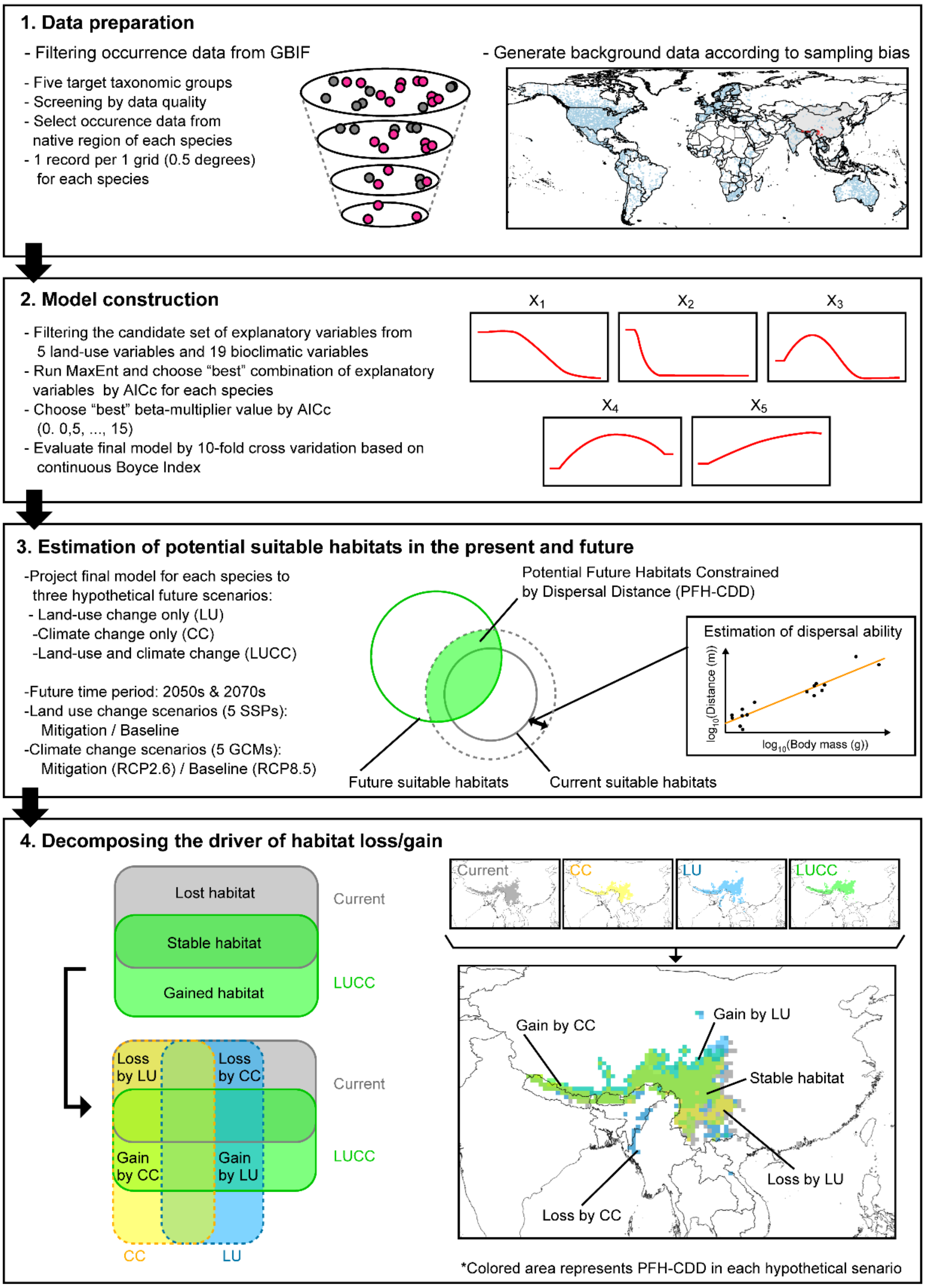

Supplementary Figure 1 Schematic diagram of modelling procedure. World maps were generated by using software QGIS ver 2.18.27 and polygon data obtained from http://www.iucnredlist.org/.

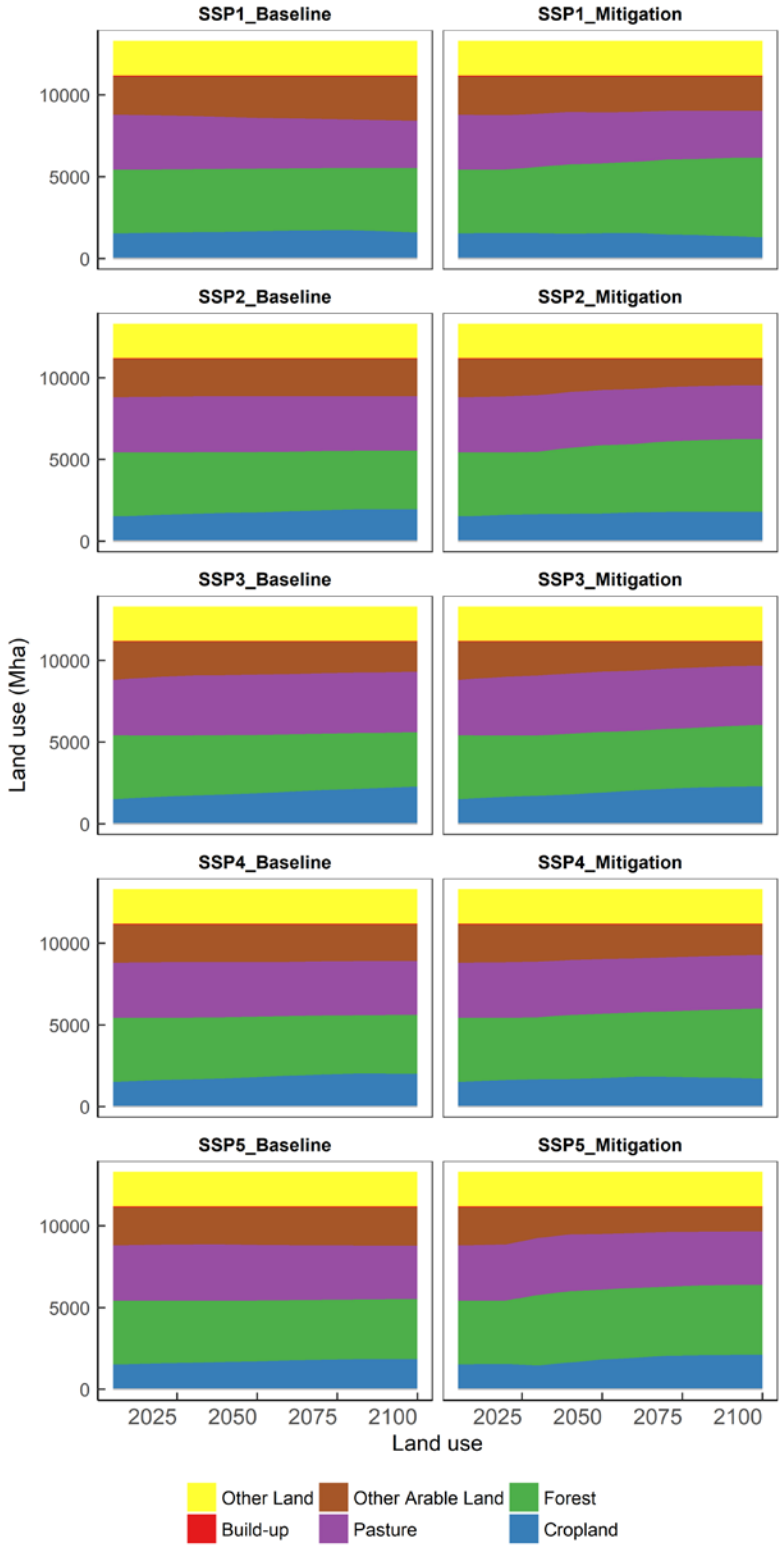

Supplementary Figure 2 Global land use change for baseline and mitigation scenarios. Source data are provided as a Source Data file.

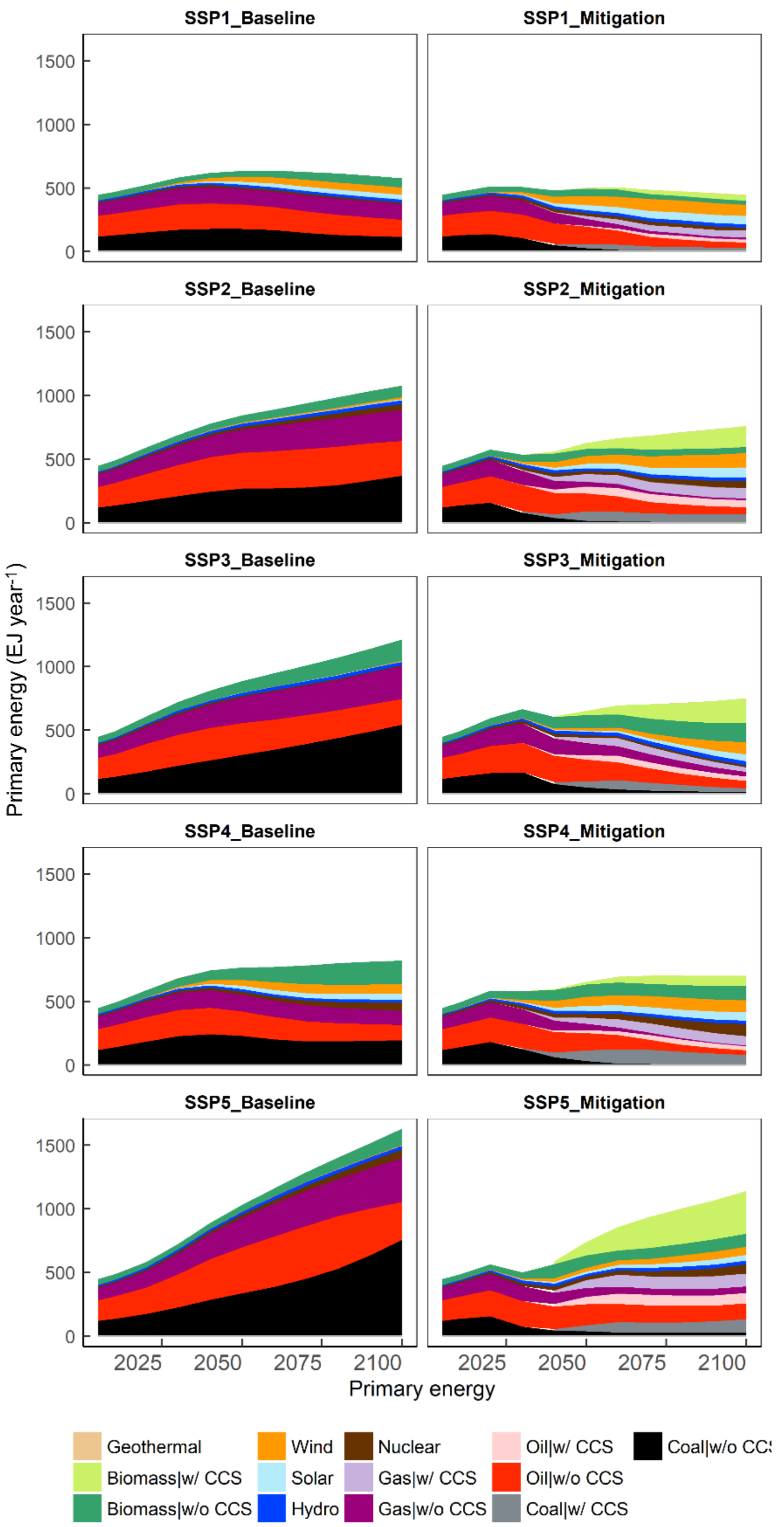

Supplementary Figure 3 Global primary energy supply for baseline and mitigation scenarios. Source data are provided as a Source Data file.

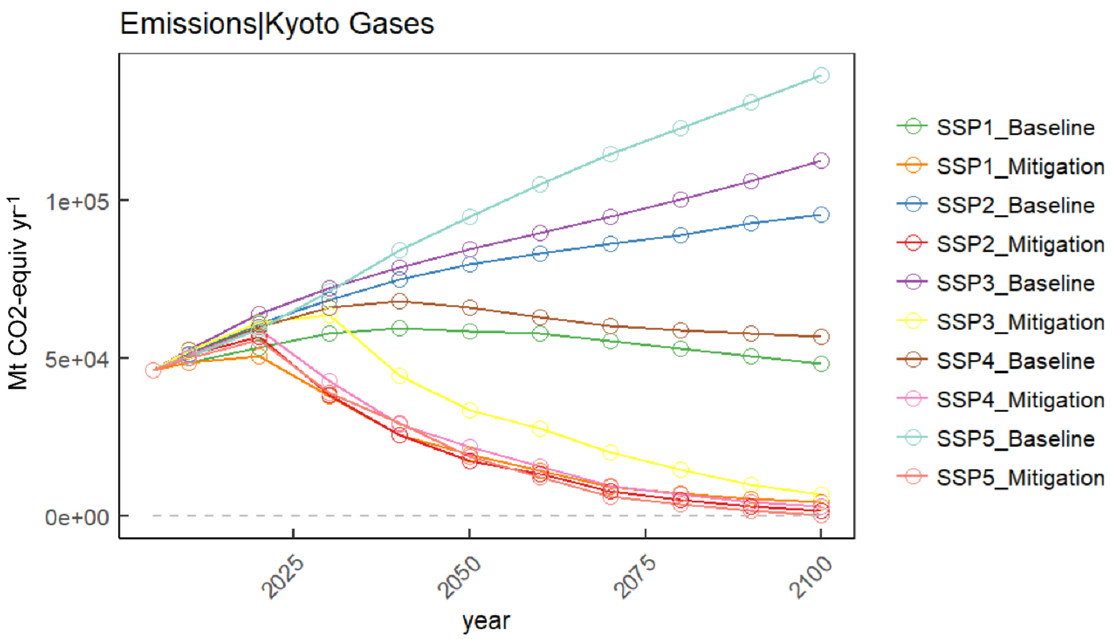

Supplementary Figure 4 Global Kyoto gas emissions for baseline and mitigation scenarios. Source data are provided as a Source Data file.

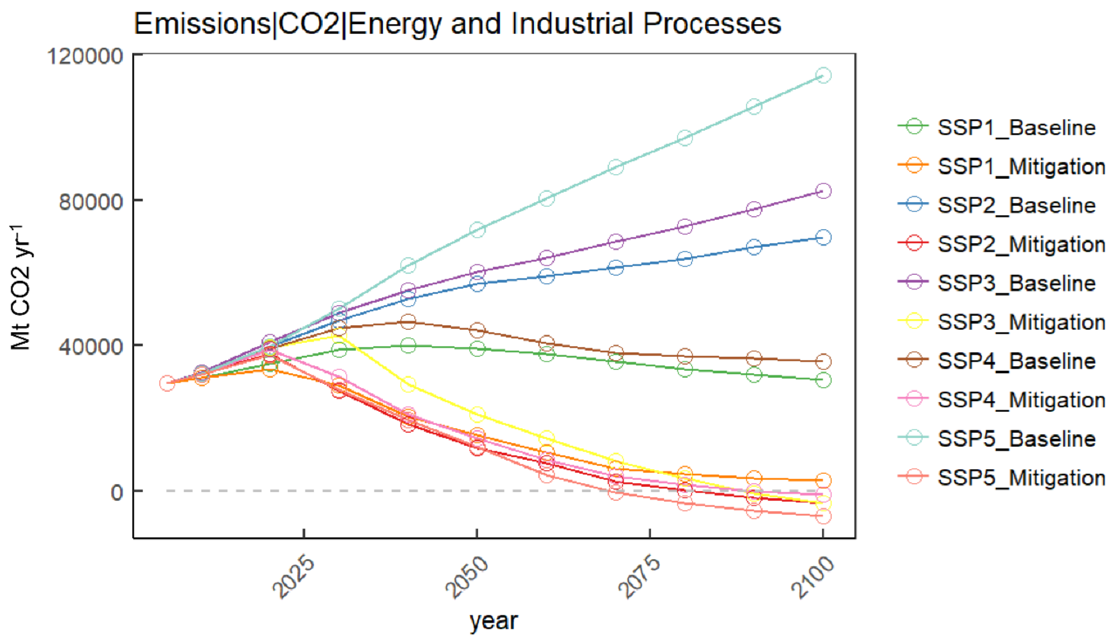

Supplementary Figure 5 Global energy and industrial processes CO_2 emissions for baseline and mitigation scenarios. Source data are provided as a Source Data file.

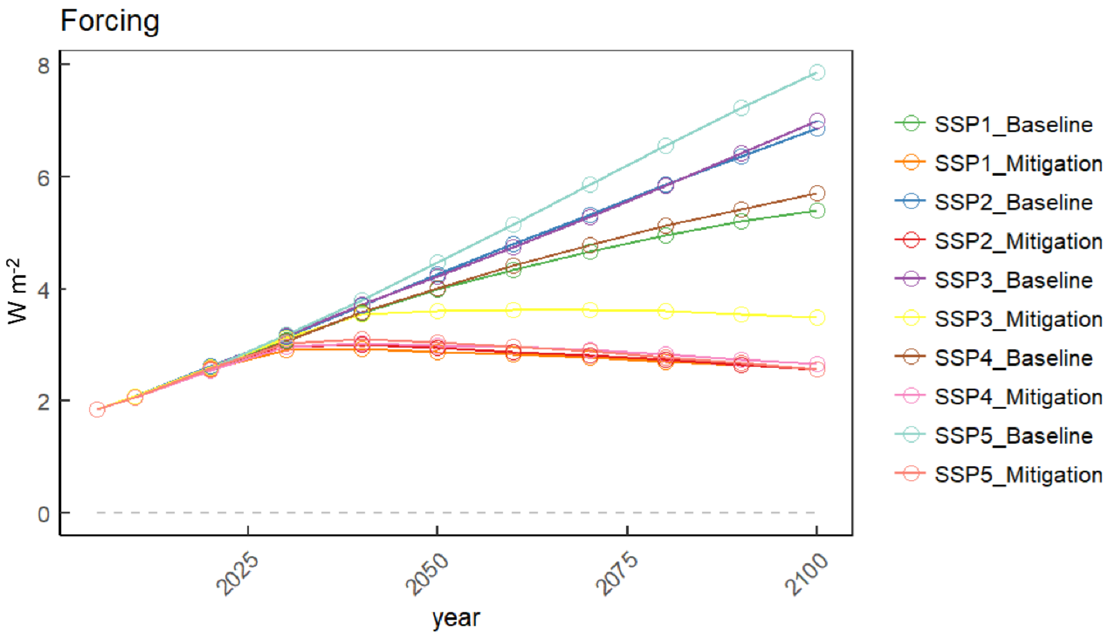

Supplementary Figure 6 Radiative forcing for baseline and mitigation scenarios. Source data are provided as a Source Data file.

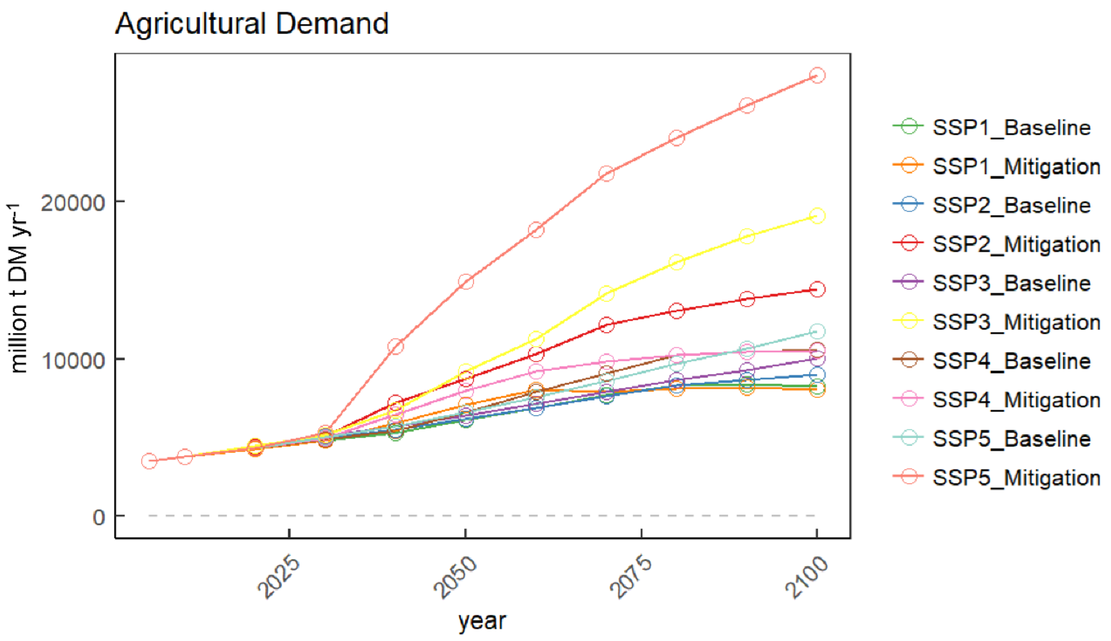

Supplementary Figure 7 Global agricultural demand (including bioenergy) for baseline and mitigation scenarios. Source data are provided as a Source Data file.

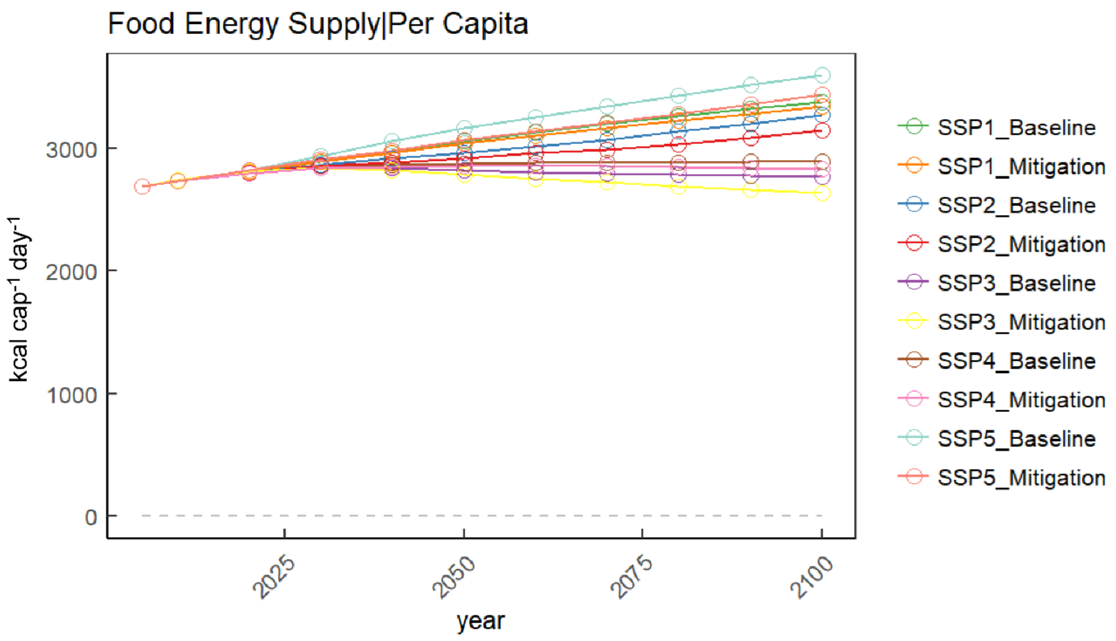

Supplementary Figure 8 Global mean food supply (per capita calorie intake). Source data are provided as a Source Data file.

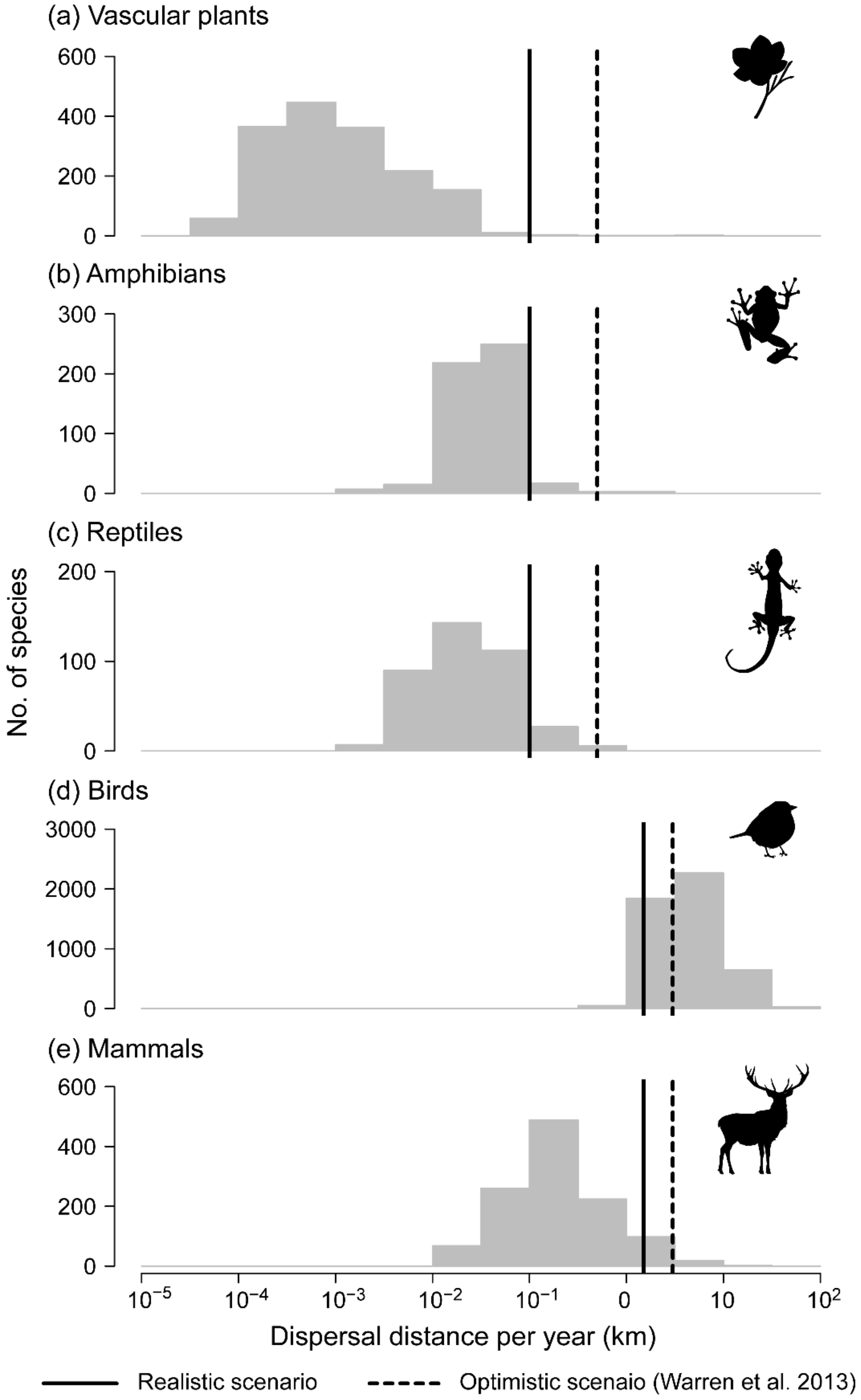

Supplementary Figure 9 Histogram of estimated dispersal distance per year, with referencing the values in realistic scenario and optimistic scenario in Warren et al. (2013)[15]. Source data are provided as a Source Data file.

Supplementary References

1. Muñoz M. C. & Thorbjarnarson, J. Movement of captive-released Orinoco crocodiles (*Crocodylus intermedius*) in the Capanaparo River, Venezuela. *J. Herpetol.* **34,** 397-403 (2000).
2. Tucker, A. D., McCallum, H. I., Limpus, C. J. & McDonald, K. R. Sex-biased dispersal in a long-lived polygynous reptile (*Crocodylus johnstoni*). *Behav. Ecol. Sociobiol.* **44,** 85-90.
3. Warner, D. A. & Shine S. Determinants of dispersal distance in free-ranging juvenile lizards. *Ethology* **114,** 361-368 (2008).
4. Dubey, S. Brown, G. P., Madsen, T. & Shine, R. Male-biased dispersal in a tropical Australian snake (*Stegonotus cucullatus*, Colubridae). *Mol. Ecol.* **17,** 3506-3514 (2008).
5 Read, J. L. Longevity, reproductive effort and movements of three sympatric Australian arid-zone geckos. *Aust. J. Zool.* **47,** 307-316 (1999).
6. Bock B. C. & McCracken G. F. Genetic structure and variability in the Green Iguana (*Iguana iguana*). *Journal of Herpetology* **22,** 316-322 (1988).
7. Olsson, M. Gullberg, A. & Tegelström, H. Determinants of breeding dispersal in the sand lizard, *Lacerta agilis*, (Reptilia, Squamata). *Biol. J. Linn. Soc.* **60,** 243-256 (1997).
8. Tokue, Y., Osawa, S. & Imamura F. A study about migration and dispersal distance of animals for ecological network planning in urban area. *J. Jpn. Soc. Reveget. Tech.* **37,** 203-206 (2011). In Japanese.
9. Sumner J., Rousset, F. Estoup, A. & Moritz C. 'Neighborhood' size, dispersal and density estimates in the prickly forest skink (*Gnypetoscincus queenslandiae*) using individual genetic and demographic methods. *Mol. Ecol.* **10,** 1917-1927 (2001).
10. Olsson, M. & Shine, R. Female-biased natal and breeding dispersal in an alpine lizard, *Niveoscincus microlepidotus. Biol. J. Linn. Soc.* **79,** 277-283 (2003).
11. Brown, W. S., Pyle, D. W., Kimberly, R. G. & Friedlaender, J. B. Movements and temperature relationships of Timber Rattlesnakes (*Crotalus horridus*) in Northeastern New York. *J. Herpetol.* **16,** 151-161 (1982).
12. Haramura, T., Yamane, M. & Mori, A. Preliminary survey on the turtle community in a lotic environment of the Kizu river. *Curr. Herpetol.* **27,** 101-108 (2008).
13. Myhrvold, N. P. et al. An amniote life-history database to perform comparative analyses with birds, mammals, and reptiles. *Ecology* **96,** 3109 (2015).
14. Meiri, S. Length-weight allometries in lizards. *Journal of Zoology* 281, 218-226 (2010).
15. Warren R. et al. Quantifying the benfit of early climate change mitigation in avoiding biodiversity loss. *Nat. Clim. Change* **3,** 678–682 (2013).
