# Supplementary material for: Biodiversity can benefit from climate stabilization despite adverse side effects of land-based mitigation
Source: Nat Commun. 2019 Nov 20;10:5240. doi: 10.1038/s41467-019-13241-y (PMC6868141; doi:10.1038/s41467-019-13241-y)
Supplement: Supplementary file 3 — Description of Additional Supplementary Information [file 41467_2019_13241_MOESM3_ESM.pdf]

Supplementary Data 1:

Basic information of modelled species and summary of model performance.
